# Tunability of Band Gap and Photoluminescence in CH_3_NH_3_PbI_3_ Films by Anodized Aluminum Oxide Templates

**DOI:** 10.1038/s41598-017-02144-x

**Published:** 2017-05-15

**Authors:** Zhan Zhang, Min Wang, Lixia Ren, Kexin Jin

**Affiliations:** Shaanxi Key Laboratory of Condensed Matter Structures and Properties, School of Natural and Applied Sciences, Northwestern Polytechnical University, Xi’an, 710072 P. R. China

## Abstract

Hybrid organic-inorganic halide CH_3_NH_3_PbI_3_ perovskite films are deposited on anodized aluminum oxide templates with the different pore diameters via one-step spin coating method. The obvious 0.082 eV blue shift of optical band gap is observed in films with decreasing the diameters of pores from 400 to 30 nm. And numerical simulations based on finite element modeling are carried out to represent the absorption edge and consistent with the experiment results. It is interesting that the films show the intense photoluminescence with the excitation intensity of less than 1 μW. Moreover, the photoluminescence intensity is increased with increasing pore diameters, which is attributed to the radiative recombination rate of photogenerated electrons and holes. These results pave a way for the further understanding of tunable photophysical properties of perovskite films.

## Introduction

During the past few years, the hybrid organic-inorganic halide perovskites solar cells (PSCs) have attracted much attention as the most ideal light absorbers because their power conversion efficiency rises from 3.8% to 22.1%^1, 2^, which is incredible in such a short time compared with other solar cells, such as c-Si, thin film CIGS, SrTiO_3_ and CdTe photovoltaic materials^3, 4^. Furthermore, these perovskites (typically, CH_3_NH_3_PbI_3_) exhibit novel and intriguing physical properties, *e. g*., large light absorption throughout the UV-Vis region, suitable band gap (~1.5 eV), small exciton binding energy (20–50 meV) and long electron-hole diffusion length (>175 μm)^5–7^. Meanwhile, a lot of efforts have been made in the crucial challenges of CH_3_NH_3_PbI_3_-based solar cells, such as substituting nontoxic Sn for toxic Pb^8^, improving the stability by hole-transporting layer and using a vacuum flash-assisted solution process for high-efficiency large-area PSCs^9, 10^. As we know, in the mesoporous configuration of hybrid organic-inorganic halide PSCs, mesoporous oxide films are usually used as scaffold layers, which are composed of semiconductor materials or insulators owing to the ability of perovskites to transport electrons as well as holes. Originally, the CH_3_NH_3_PbI_3_ perovskites were prepared by spin-coating onto the mesoporous TiO_2_ to form a dye-sensitized liquid solar cell in 2009^1^. Since then, mesoporous oxide materials have been usually used as scaffold layers to transport electrons and holes. In 2012, Snaith *et al*. replaced the *n*-type mesoporous TiO_2_ with insulating mesoporous Al_2_O_3_ in PSCs, revealing that perovskites can be used not only as sensitizers but also as electron and hole transport layers^11^. So far, insulating Al_2_O_3_ materials have been widely used in PSCs. Different kinds of mesoporous scaffolds have an impact on final properties of perovskites and a high degree of preferential orientation has been detected for Al_2_O_3_ scaffolds^12^. Moreover, the long-term stability of PSCs would be improved with a porous Al_2_O_3_ buffer layer or ultrathin Al_2_O_3_ layers prepared by atomic layer deposition^13, 14^. Nevertheless, there is a special Al_2_O_3_ substrate named anodized aluminum oxide (AAO) template, which is considered to be a very useful and promising material for applications in nanotechnology because of its penetrable and highly ordered porous structure. To this day, most nanowires, nanotubes and nanoparticles have been produced successfully using AAO templates^15^. Furthermore, flexible and mechanically robust PSCs have been fabricated on plastic substrates with inverted nanocone AAO structures^16^.

On the other hand, the tunable band gap is an important feature of nanocrystals, *i. e*., band gap engineering. As we have already known, the band gap of CH_3_NH_3_PbI_3_ can be adjusted by substituting Cl^−^/Br^−^ for I^− ^^17^, Sn^2+^ for Pb^2+^ or replacing CH_3_NH_3_^+^ with other organic cations^8, 18^. Moreover, different size of particles or nanocrystallization can also modulate the band gap. Recently, Demchyshyn *et al*. have utilized AAO nanotubes with diameters of 6–8 nm to shift the photoluminescence peak by 62 nm on account of quantum size effect^19^. In addition, Lee *et al*. have probed crystal evolution and stability of CH_3_NH_3_PbI_3_ on AAO templates with different pores in diameter^20^. Thus, it is still significant to explore their photophysical properties by changing pore diameters although the films have been prepared on AAO templates mentioned above. In this case, we deposit CH_3_NH_3_PbI_3_ films via one-step spin coating method on AAO templates with the penetrable and high-ordered pores ranging from 30 to 400 nm in diameter (*d*_*pore*_). And the structural and photophysical properties have been investigated primarily in order to have a better understanding of intrinsic mechanisms in the CH_3_NH_3_PbI_3_/AAO nanostructures, which exhibit the blue shift of band gap and the reduced photoluminescence with decreasing the size of pores.

## Results and Discussion

**Figure 1:**
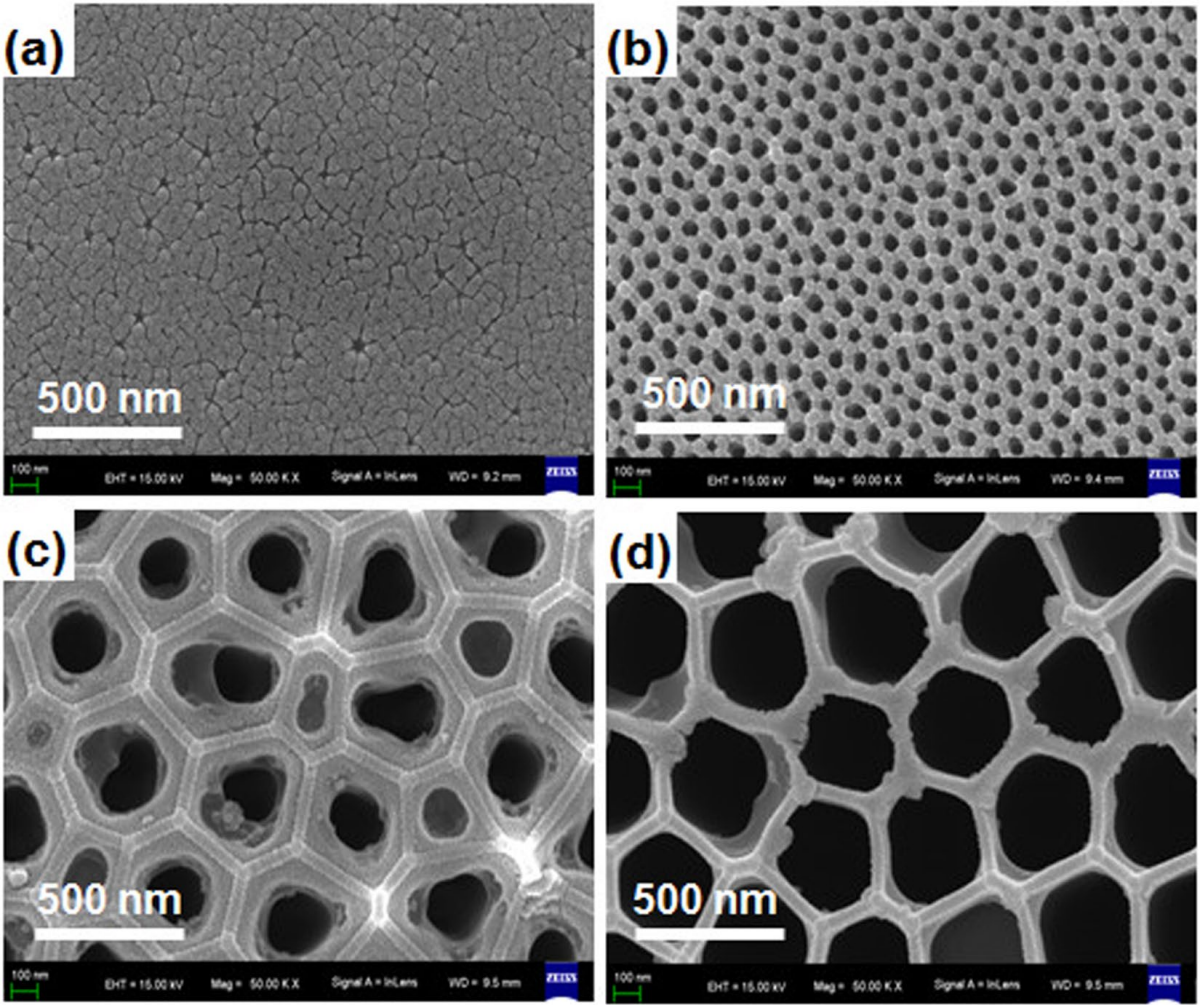
Top-down SEM images of the perovskite films on AAO templates with different pores in diameter: (**a**) 30, (**b**) 60, (**c**) 200, (**d**) 400 nm. In all images, scale bars are 500 nm.

The SEM images of the top surface and cross-section morphologies of CH_3_NH_3_PbI_3_ on AAO templates with different pore diameters are shown in Fig. 1 and Fig. S1, respectively. We can see that all CH_3_NH_3_PbI_3_ films have discontinuous crystal grains. The film on the AAO template with *d*_pore_ = 30 nm covers the great majority of templates and produce a lot of gaps among the grains with the size of about 40 nm, compared with the pure templates (Fig. S2). The grain size of CH_3_NH_3_PbI_3_ film on the AAO template with *d*_*pore*_ = 60 nm is about 60 nm. It is difficult to determine the accurate grain size for the films on the AAO templates with larger *d*_*pore*_. In addition, it is obvious that the CH_3_NH_3_PbI_3_ films firstly crystallize on hexagonal edges of AAO templates and the pores are not fully filled. For cross-section morphologies, the crystallization of films mainly takes place along the walls of holes. The optical images of the pure AAO template and the template after depositing the film are shown (Fig. S3). We can see that the pure AAO template is transparent with slightly yellow in color. After the film deposition, both the top and bottom surfaces become very black, also demonstrating that the AAO templates are well infiltrated with the solutions.

**Figure 2:**
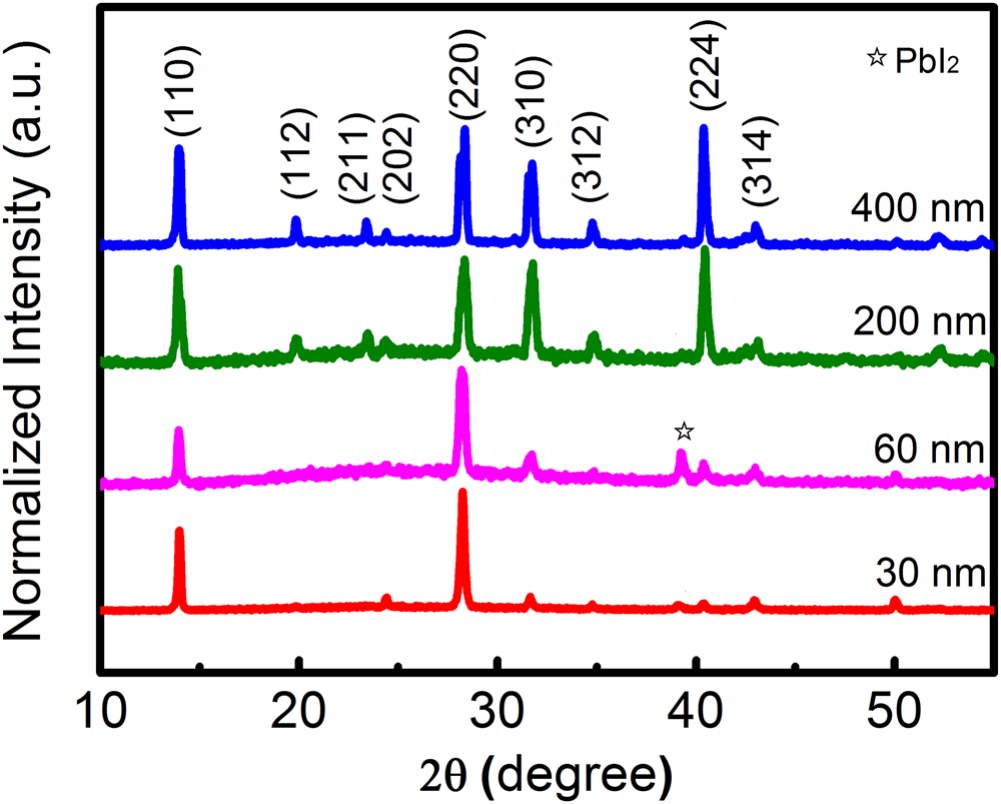
Comparison of XRD patterns of the perovskite films on AAO templates with different pores in diameter.

Figure 2 presents XRD patterns of CH_3_NH_3_PbI_3_ films fabricated on AAO templates with different pore diameters. The strong diffraction peaks at 13.9°, 28.2°, 31.8° and 40.4° can be respectively assigned to (110), (220), (310) and (224) diffractions, indicating the formation of crystalline CH_3_NH_3_PbI_3_^21^. In addition, the XRD patterns of CH_3_NH_3_PbI_3_ films on AAO templates with *d*_*pore*_ = 200 and 400 nm exhibit stronger (310) and (224) diffractions compared with those with *d*_*pore*_ = 30 and 60 nm, which mainly show strong (110) and (220) diffractions. This indicates that different pore diameters of AAO templates would affect the crystal orientation of films to a certain extent^10^. Diffraction peak at 39.2° is attributed to PbI_2_, which is not observed in CH_3_NH_3_PbI_3_ film prepared on Al_2_O_3_ single crystal substrate (Fig. S4a). And this diffraction peak of PbI_2_ on AAO template with *d*_*pore*_ = 60 nm is obvious. To further investigate this, we prepare other two batches of films. The first batch of films is prepared under the same condition mentioned above and the second is prepared by dipping the AAO templates in the same solution for 2 hours instead of the spin-casting method. The XRD patterns show the same results in the first batch of films (Fig. S4b). And the XRD patterns of the second batch of films show ultra-strong diffraction peak of PbI_2_ for all films and it is still stronger for CH_3_NH_3_PbI_3_ film on AAO template with *d*_*po*re_ = 60 nm than others (Fig. S4c). So we deduce that this phenomenon might come from the porous structure and is related to the ratio between pore and grain sizes.

**Figure 3:**
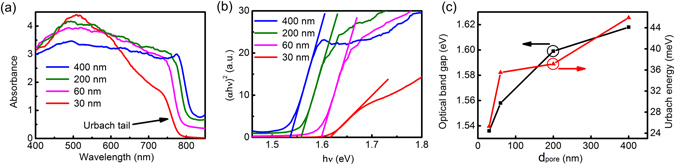
(**a**) UV-vis absorption spectrum of the films on AAO templates with different size of pores; (**b**) Tauc plot according to the absorption coefficient to estimate the band gap of the perovskite films; (**c**) Optical band gaps and Urbach energies of the perovskite films.

**Figure 4:**
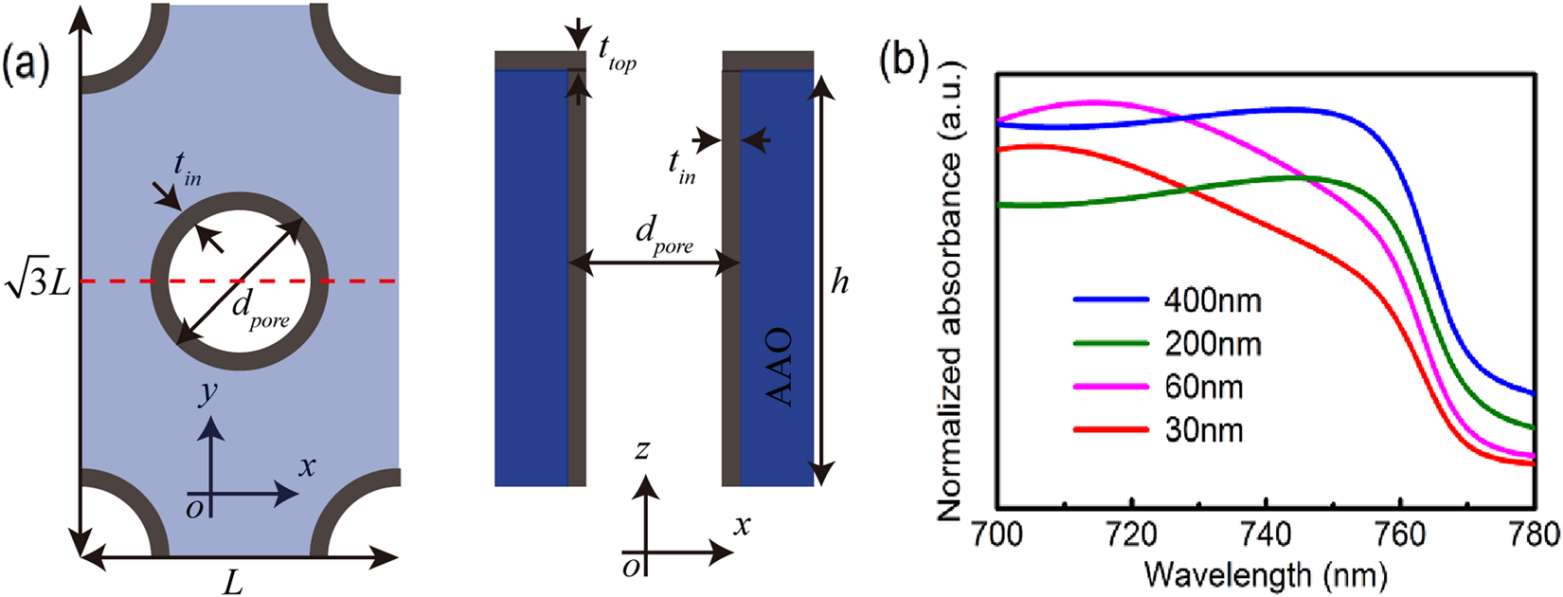
(**a**) Schematic diagram of finite element modeling. The dark, wathet blue and dark blue regions represent CH_3_NH_3_PbI_3_ on the top surface, inwall and AAO template itself, respectively; (**b**) Simulated absorbance in the range of 700~800 nm.

Figure 3a presents the UV-vis absorption spectrum of CH_3_NH_3_PbI_3_ films. High absorbance is obtained in the visible region for all the films on AAO templates. The absorption spectrum of the pure AAO templates is shown in Fig. S5. The pure AAO templates exhibit low absorption, indicating the high absorbance originates from CH_3_NH_3_PbI_3_ films. According to the absorption coefficient, a tauc plot of (*αhν*)^2^-*hν* is used to determine the optical band gap, as shown in Fig. 3b. And the relationship between *d*_*pore*_ and optical band gap is described in Fig. 3c. The optical band gap is increased from 1.536 eV to 1.618 eV with decreasing *d*_*pore*_ of AAO templates from 400 nm to 30 nm. Thus an obvious 0.082 eV blue shift of optical band gap is produced. In addition, the Urbach energy of the CH_3_NH_3_PbI_3_ films can be obtained by fitting the absorbance curve at the optical band edge using an empirical equation^22^. As shown in Fig. 3c, the Urbach energy is proportional to the *d*_*pore*_ of AAO templates. It is known that Urbach energy is a characterization of structural disorder in the materials and higher Urbach energy is related to higher disorder. Hence, it indicates larger pores of AAO templates lead to higher disorder in CH_3_NH_3_PbI_3_ films. In general, there are two mechanisms giving rise to the blue shift, including the doping effect and the quantum size effect. Obviously, the doping effect can be excluded in our work. Then the quantum size effect generally would affect the band gap. However, Bohr radius of CH_3_NH_3_PbI_3_ films is about 2.2 nm calculated from the magnetoabsorption spectrum^23^, and the minimum grain size obtained from the SEM patterns in our work is ~40 nm on AAO template with *d*_*pore*_ = 30 nm. Thus the contribution of quantum size effect to the blue shift of our films is negligible, which is completely different from the results investigated by Demchyshyn *et al*.^19^. Thereby, based on finite element modeling, we carry out the investigation on numerical simulations to represent the absorption feature in wavelength range from 700 to 780 nm. The model is shown in Fig. 4a. The main physical parameters of *t*_*in*_, *t*_*top*_, *d* denote the thickness of films adhered on the inwall of the pore, the thickness of films deposited on AAO top surface, and the wall thickness of AAO, respectively. These values are estimated from the SEM images for different cases, and increasing *t*_*in*_ and decreasing *t*_*top*_ are set with increasing the *d*_*pore*_ of AAO templates (Table S1). By considering the higher order degree of the top surface and the capability of the computer, the height of AAO template is set as 1 μm. Simulation results are shown in Fig. 4b. It is clear that the whole tendency of the absorption curve agrees well with the experimental data. While the degree of the blue shift is smaller than the experimental value, which seems to result from the dielectric constant of single crystal is used in our calculation^24^. When the pore size is small and the period is short, the dispersive relationship of the structure can be equaled as a homogeneous material based on Maxwell-Garnett effective medium method. The absorption behavior can be calculated by Snell’s low^25^. For the long period, the model can be regarded as a dielectric waveguide^26^. The cutoff wavelength is in direct proportion to the *d*_*pore*_. Thus, the absorption edge is blue-shifted as the *d*_*pore*_ decreases. In additional, the minimal absorbances are different. It is because that the probability of photons coming into the waveguide becomes larger as the *d*_*pore*_ increases. The incoming photons are absorbed during propagation in the pore.

**Figure 5:**
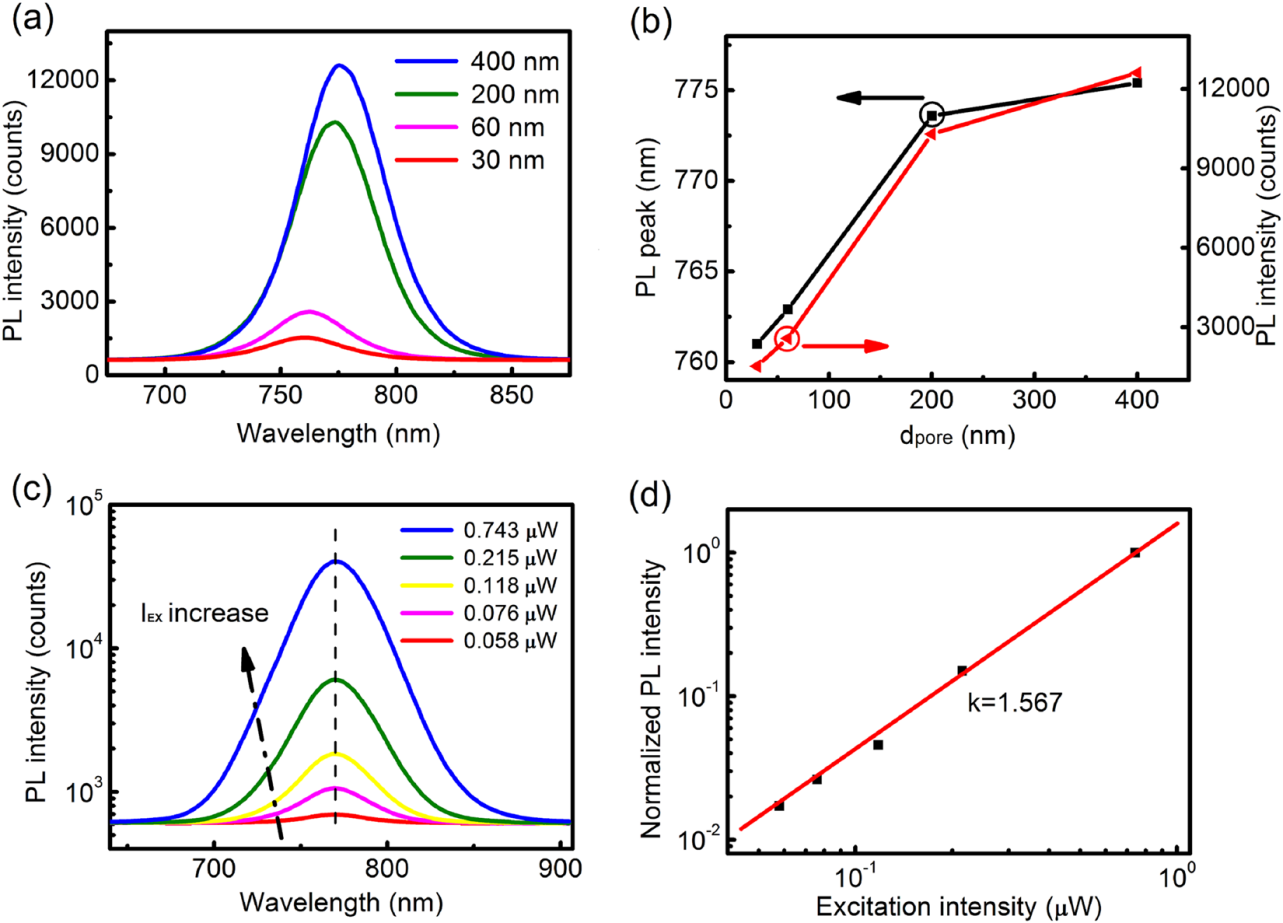
(**a**) PL spectra of the perovskite films on AAO templates with different size of pore; (**b**) Emission peaks and intensities of the perovskite films; (**c**) PL spectra of the perovskite film on AAO template with d_pore_ = 200 nm recorded with excitation intensity from 0.058 to 0.743 uW. All spectra are measured in air at room temperature. The peak energy is indicated by the dashed line; (**d**) Logarithm plot of the integrated PL intensity versus excitation intensity.

**Figure 6:**
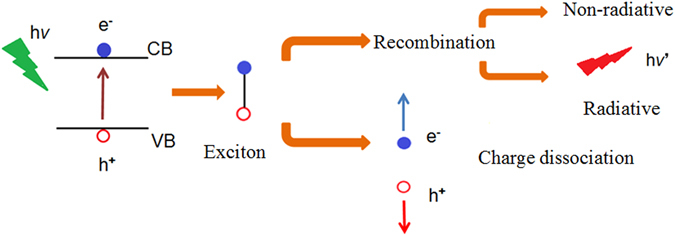
Schematic diagram based on the band theory shows that electron-hole pair has two opposite ways: charge dissociation or recombination (radiative and non-radiative recombination).

Figure 5a presents the photoluminescence (PL) spectrum of CH_3_NH_3_PbI_3_ films on AAO templates with different pore diameters with a constant excitation intensity of 0.3 μW. The PL peaks as shown in Fig. 5b are consistent with that of the UV-vis peaks in Fig. 3b, which has a 14 nm blue-shift from 775 nm to 761 nm with decreasing *d*_*pore*_ of AAO templates. The PL intensity exceeds 10^4^ counts with the excitation intensity less than 1 μW. Moreover, the PL intensities are increased with increasing pore diameters of AAO templates. To further examine the relationship between PL intensity (*I*_PL_) and excitation intensity (*I*_EX_), we measure the PL intensity of CH_3_NH_3_PbI_3_ films on AAO template with *d*_*pore*_ = 200 nm at the excitation intensity ranging from 0.058–0.743 μW in Fig. 5c. When the excitation power intensity is higher than 0.75 μW, the PL intensity of the CH_3_NH_3_PbI_3_ film would not be determined by our optical spectrometer system due to the saturation. In direct bandgap semiconductors under non-resonant excitation conditions, the *I*_PL_ is a power-law function of the *I*_EX_, which is expressed by: *I*_PL_∝*I*_EX_^*k*^, where 1 < *k* < 2 for recombination of excitons^27^. Figure 5d shows the PL intensity vs. excitation intensity on a double-logarithmic scale. We obtain a power-law exponent *k* of 1.567, which is very close to that for excitons of CH_3_NH_3_PbI_3_ films on planar glass substrates^28, 29^. The intrinsic mechanism of this process is the photoneutralization of the donors/acceptors, which result in competitive recombination channels^28^. Accordingly, the CH_3_NH_3_PbI_3_ film deposited on AAO template with *d*_*pore*_ = 200 nm has negligible effect on the choice of recombination channels. Furthermore, we attempt to measure the electrical property in plane and out-of-plane of all CH_3_NH_3_PbI_3_ films on AAO templates under the same irradiation, whereas the current can not be determined due to the high resistivity. Furthermore, we draw a schematic diagram based on the band theory as shown in Fig. 6. For CH_3_NH_3_PbI_3_ films, the electrons in the valence band acquire enough energy to reach the conduction band and leave holes in the valance band, forming the electron-hole pairs under the irradiation. Snaith *et al*. have considered that the morphology has essential influence to the performance of CH_3_NH_3_PbI_3_-type solar cell^30^. Generally, the electron-hole pairs would generate two opposite outcomes: generating free carriers through charge dissociation or recombination (including radiative recombination and non-radiative recombination). In our study, CH_3_NH_3_PbI_3_ films have discontinuous grain domains, which limit the electron-hole pairs to be free charges by charge dissociation. Thus, electrical properties are difficult to measure due to the limit of equipments. On the contrary, the electron-hole pairs emit photons by radiative recombination, resulting in the enhanced PL intensity exceeding 10^4^ counts with the excitation intensity less than 1 μW. Moreover, the increasing PL intensity with increasing pore diameters of AAO templates might originate from the higher disorder of films indicated through Urbach energy mentioned above. This is similar with results in the porous TiO_2_ with different pore sizes derived from the radiative recombination rate of photogenerated electrons and holes^31, 32^. Meanwhile, the *k* value obtained above demonstrates that our CH_3_NH_3_PbI_3_ films based on AAO templates have negligible effect on the choice of recombination channels^28^. In other word, the increase of radiative recombination is caused by the decrease in the charge dissociation instead of the change between radiative recombination and non-radiative recombination. The strong PL intensity has promising applications in light emitting devices and laser with changeable light-emission positions and intensity.

## Conclusion

In summary, we have detailed the structural and photophysical properties of CH_3_NH_3_PbI_3_ films fabricated on AAO templates with the pores in diameter ranging from 30 to 400 nm. Both UV-vis absorption spectrum and PL spectrum show the blue shift with decreasing pore sizes. The numerical simulations based on finite element modeling to represent the absorption edge agree well with the experiment results. And a strong linear power-law is observed in PL spectrum. In general, our work on CH_3_NH_3_PbI_3_/AAO indicate the band gap of CH_3_NH_3_PbI_3_ films can be tuned easily by changing the pore sizes of AAO templates, and the intense PL intensity can also be controlled readily, which can be utilized in light-emitting diode and laser.

## Methods

### Film preparation

CH_3_NH_3_I (0.163 g, 99.5% purity) was mixed with PbI_2_ (0.471 g, 99.99% purity) in anhydrous N,N-dimethylformamide (1 mL) by ultrasonic shaking at 60 °C for 2 hours to produce a CH_3_NH_3_PbI_3_ solution with concentration of 40 wt%. The CH_3_NH_3_PbI_3_ solution was then dropped onto an AAO template (substrate diameter 12 mm). After a 20 s delay time, the template was spun cast at 5000 rpm for 40 s. The sample was then dried on a hot plate at 120 °C for 25 min. The CH_3_NH_3_PbI_3_ solution was also spun cast on an Al_2_O_3_ substrate (substrate area 5 mm × 5 mm) under the same preparation condition as a reference. The whole process was in a nitrogen-filled glovebox.

### Structure characterization

The surface and cross-section (fractured) morphology of the thin films were characterized by using a scanning electron microscope (SEM) (JSM-6700F, JEOL). X-ray diffraction (XRD) was performed using a X-ray diffractometer (XRD-7000, Shimadzu) with Cu-Kα radiation source (λ = 1.5406 Å) at a step size of 0.02°.

### Photophysical characterization

The ultraviolet-visible (UV-vis) absorption spectrum was measured using an ultraviolet-visible spectrophotometer (U-3010, Hitachi). The photoluminescence (PL) spectrum of the thin films was performed with an optical spectrometer (SP-2500, Princeton Instruments) using a 532-nm radiation pulsed laser beam.

## Electronic supplementary material

Supplementary Information
