# Supplementary material for: Tunability of Band Gap and Photoluminescence in CH3NH3PbI3 Films by Anodized Aluminum Oxide Templates
Source: Sci Rep. 2017 May 15;7:1918. doi: 10.1038/s41598-017-02144-x (PMC5432502; doi:10.1038/s41598-017-02144-x)
Supplement: Supplementary file 1 — Supplementary Information [file 41598_2017_2144_MOESM1_ESM.doc]

**Supplementary Information**

**Tunability of Band Gap and Photoluminescence in CH_3_NH_3_PbI_3_ Films by Anodized Aluminum Oxide** **Templates**

Zhan Zhang, Min Wang, Lixia Ren & Kexin Jin*

Shaanxi Key Laboratory of Condensed Matter Structures and Properties, School of Natural and Applied Sciences, Northwestern Polytechnical University, Xi’an, Shannxi, 710072, P. R. China

*E-mail: jinkx@nwpu.edu.cn


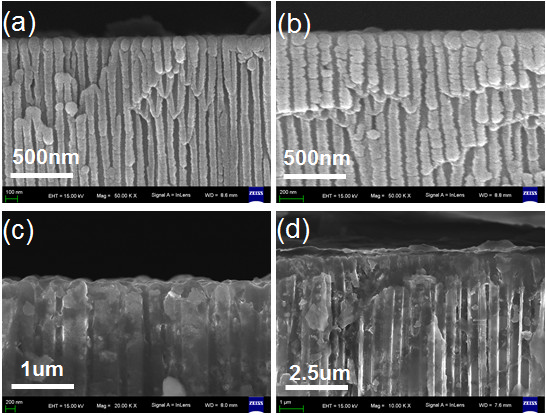


**Figure S1.** Cross-section SEM images of the CH_3_NH_3_PbI_3_ perovskite films on AAO templates with different pores in diameter: (a) 30, (b) 60, (c) 200, (d) 400 nm.


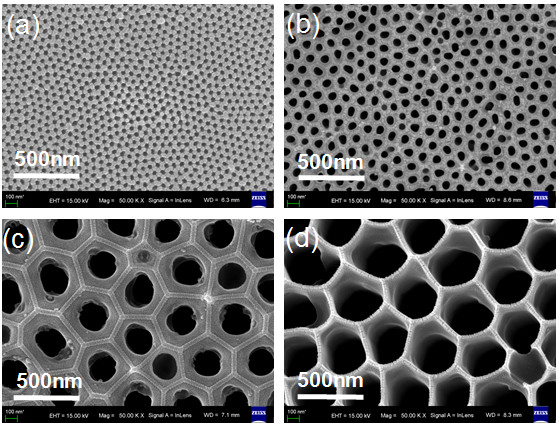


**Figure S2.** Top-down SEM images of the pure AAO templates with different pores in diameter: (a) 30, (b) 60, (c) 200, (d) 400 nm.


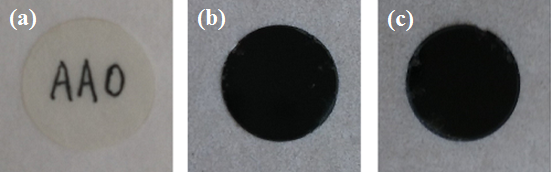


**Figure S3.** Optical images of (a) the pure AAO template placed on a piece of white paper marked *“AAO”*; (b) top and (c) bottom surface of the CH_3_NH_3_PbI_3_/AAO template after thermal annealing. (*d_pore_*=200 nm)


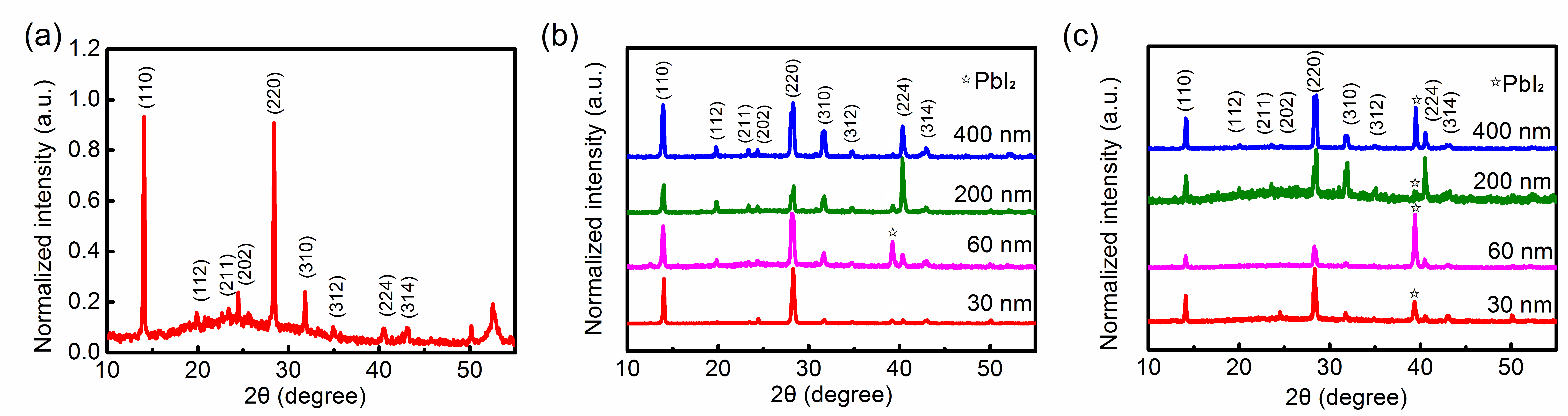


**Figure S4.** XRD patterns of (a) CH_3_NH_3_PbI_3_ film on Al_2_O_3_ substrate; (b) control groups of the (b) first and (c) second batch of CH_3_NH_3_PbI_3_ perovskite films on AAO templates with different pores in diameter.


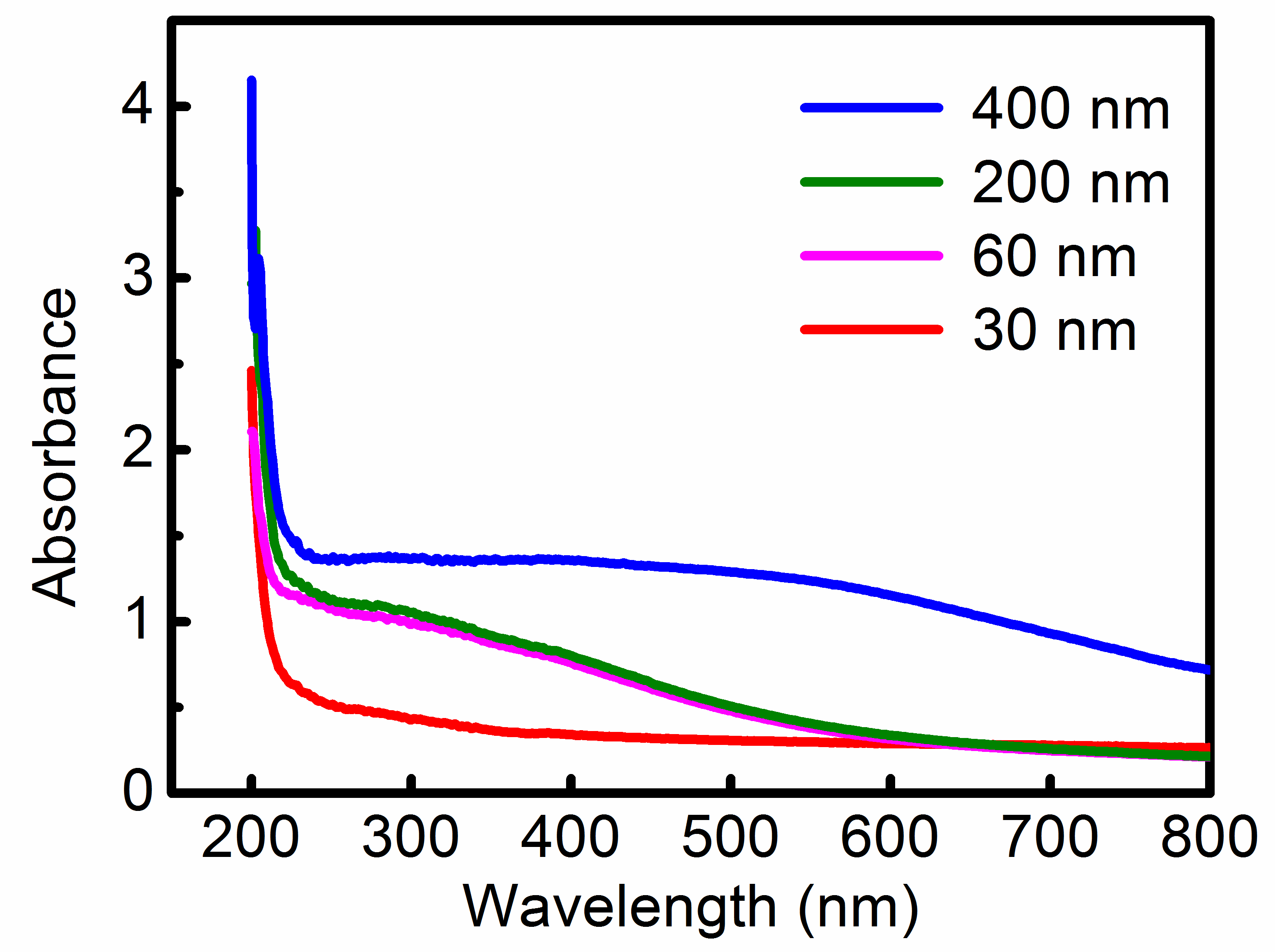


**Figure S5.** UV-vis absorption spectrum of the pure AAO templates with different size of pores.

**Table S1.** Values of *d_pore_*, *t_in_*, *t_top_*, *d* in finite element modeling. (nm)

| *d_pore_* | *t_top_* | *t_in_* | *d* |
| --- | --- | --- | --- |
| 30 | 70 | 10 | 20 |
| 60 | 60 | 20 | 30 |
| 200 | 60 | 30 | 80 |
| 400 | 50 | 50 | 100 |
